# Tracheotomy with the Thermo-Cautery

**Published:** 1879-06-20

**Authors:** P. S. Conner


### *TRACHEOTOMY WITH THE THERMO-CAUTERY.*

BY P. S. CONNER, M.D.

On the 15th February last, with the kind assistance of my friends, Drs. Thornton and Forchheimer, I made tracheotomy in a case of diphtheritic croup, operating with the Paquelin thermo-cautery. The case had been under Dr. Thornton's care for but four or five hours, though the child had been sick for several days. When I saw it there was a decidedly cyanotic appearance of the face, the pulse was rapid and feeble, and the breathing almost entirely diaphragmatic, the epigastric change of level being greater than I had ever before seen. Extensive diphtheritic patches and ulcerations were visible in the throat. Notwithstanding the appearent hopelessness of the case, tracheotomy was deemed advisable. The child having been thoroughly etherized, the anæsthetic was removed, and the soft parts down to the trachea divided with the thermo-cautery knife.

So far the operation was absolutely bloodless. The trachea having been thus exposed, the parts were fixed by a tenaculum, and section of the upper rings made with and ordinary bistoury, considerable hemorrhage resulting. Very soon afterwards in a paroxysm of coughing a large detached glutinous cast about 1½ inch long and from ¼ to ½ inch in diameter was brought up into the opening, from which I readily removed it. The color of the face and lips rapidly improved, and the respiration became almost normal. An ordinary double canula was introduced, and the child left in the care of its parents, who were directed to keep the tube clear during the night. Dr. Thornton informs me that at 8 o'clock the next morning he was called to see the child, apparently dying, and found the canula blocked up with the exceedingly viscid secretion that is so constantly met with in these cases. Having removed the tubes, and by artificial respiration re-established the proper breathing, the tubes were thoroughly cleaned and reintroduced. The difficulty of keeping them free, however, was so great that early in the afternoon their use was abandoned. The child, I am told, went along comfortably until near 8 p. m., when it began to rapidly sink and died in a few minutes, having lived about 21 hours after the operation.

The only point of special interest in the case, of course, is the use of the thermo-cautery, which worked admirably. Though there is a wide diversity of opinion among the French surgeons, who most of all have used it, as to the advisibility of its employment in these operations upon the wind-pipe, still it seems to me that the instrument must prove of great service; enabling us as it does, certainly in the great majority of cases, to avoid the trouble and danger consequent upon loss of blood. Take away the fear of hemorrhage, and no properly instructed man, I care not how unaccustomed to operating he might be, would hesitate to make tracheotomy. That any bloodless method will make much difference in the mortality is very questionable, for patients ordinarily die not of the operation but of the disease. If operative procedures are to have any special influence upon the disease they must be instituted early, not when the patient is in *extremis*. Perhaps for the reason already given physicians will be more ready to urge and patients to consent to an early operation if it is to be made with the thermo-cautery or galvano-cautery. If not made early, shall tracheotomy be made at all in cases of croup and diphtheria? Certainly, it will unquestionably now and then save a case that otherwise would be lost, and, whatever the result, it will accomplish one great good if no other. It will enable the patient to live comfortably while he does live, and die quietly; in a word, secure *euthanasia*, death coming "after the fashion and semblance of a kindly and pleasant sleep."—*Lancet and Clinic.*

